# Quantitative expression of osteopontin in nasal mucosa of patients with allergic rhinitis: effects of pollen exposure and nasal glucocorticoid treatment

**DOI:** 10.1186/1710-1492-6-28

**Published:** 2010-11-02

**Authors:** Serena E O'Neil, Carina Malmhäll, Konstantinos Samitas, Teet Pullerits, Apostolos Bossios, Jan Lötvall

**Affiliations:** 1 Krefting Research Centre, Sahlgrenska Academy, University of Gothenburg, Sweden; 2 7th Respiratory Department, Athens Chest Hospital ("Sotiria"), Greece

## Abstract

**Background:**

Osteopontin (OPN) is a multifunctional cytokine that has been primarily investigated in Th1 diseases. Recently, it has also been implicated in Th2-mediated allergic diseases, such as asthma. The expression of OPN in allergic rhinitis (AR) is currently unknown, as is the effect of intranasal glucocorticosteroids (GCs) on that expression.

**Methods:**

Subjects with AR were randomised to receive treatment with fluticasone propionate (FP) (n = 12) or a placebo (n = 16) over the grass pollen season and nasal biopsies were taken prior to, and during the season. OPN expression in the nasal mucosa was examined with immunohistochemistry. Healthy non-AR controls (n = 5) were used as a comparator.

**Results:**

OPN expression was detected in epithelial cells, subepithelial infiltrating/inflammatory cells and cells lining the vessels and glands of all subjects. Comparison of the pre- and peak-pollen season biopsy sections in placebo treated patients revealed no increase in OPN expression during the grass pollen season (5.7% vs 6.4%). Treatment with a local glucocorticosteroid did not alter the expression of OPN during pollen exposure (6.2% vs 6.7%).

**Conclusion:**

OPN has been increasingly associated with the pathogenesis of various Th2-mediated diseases. However, our finding that the OPN expression in the nasal mucosa of AR patients is not significantly affected by allergen exposure and is comparable to that of the healthy controls, suggests that intracellular OPN is not directly involved in the pathogenesis of allergic rhinitis.

## Background

The inflammatory process in allergic rhinitis (AR) involves many different inflammatory cells, cytokines, chemokines and other regulatory molecules [1]. It is well known that exposure to allergens, including natural pollen exposure, primarily enhances eosinophilic inflammation in the nose [2] and increases cytokine release [1]. Furthermore, local glucocorticoids are efficient in attenuating the allergen-induced inflammation and the cytokine expression, as we and others have documented in nasal mucosal studies [2-6].

OPN is a pleiotropic cytokine normally expressed by many cell types [7], which has been implicated in various diseases [8], including asthma [9-11] and chronic rhinosinusitis [12]. OPN can be expressed in eosinophils, which could argue its involvement in allergic eosinophilic inflammation [13]. Studies of OPN expression have quantified the expression in different ways, including concentrations in lavage fluid, the expression of OPN mRNA by RT-PCR and the semi-quantification of cells expressing OPN by immunohistochemistry.

The aim of the current study was to determine the level and tissue distribution of OPN expression in the nasal mucosa of patients with AR and to determine whether OPN expression is affected by allergen exposure during a grass pollen season in allergic individuals. We also aimed to investigate whether a nasal glucocorticoid affected local OPN expression. The nasal biopsies used in the current study have previously been evaluated in other studies showing clear clinical effects of pollen exposure, as well as effects of a nasal glucocorticoid treatment on both symptoms of rhinitis, eosinophilia and expression of several cytokines [2,5].

## Materials and methods

Nasal biopsy samples were obtained as a part of a previously published clinical study [2]. Briefly, grass pollen sensitised allergic rhinitis (AR) subjects were randomised to receive the intranasal glucocorticocoid, fluticasone propionate (FP) (200 μg/day) (n = 12; median age 30.5, range 18-40 years) or placebo (n = 16; median age 30, range 16-48 years) for the duration of the grass pollen season, starting approximately 2 weeks before the expected onset. The nasal biopsies were collected 1-2 months before the commencement of treatment and at the peak of the season. Ethics approval was obtained from the Ethics Review Committee of Clinical Research Studies at the University of Tartu, Estonia. Written informed consent was provided by all participants. As controls, nasal biopsies were taken from five healthy, non-allergic individuals prior to the pollen season [5].

Nasal biopsy sections were immersed in OCT compound in cryomoulds (Tissue-Tek, Sakura Finetek Europe, Zoeterwouede, Netherlands), snap frozen in liquid nitrogen and stored at -80°C prior to processing. Tissue sections of 5 μm thickness were prepared, wrapped in aluminium foil and stored at -80˚C. Thawed sections were fixed in 2% formaldehyde and treated with pre-heated PBS containing 0.0064% sodium azide, 0.18% glucose, 0.1% saponin, 1:3750 glucose oxidase, for 40 mins to inhibit endogenous peroxidise. Unspecific binding was blocked for 30 mins using 10% rabbit serum (DAKO Denmark A/S, Denmark). Sections were incubated with mouse anti-osteopontin monoclonal antibody (clone 190312. MAB1433) (R&D Systems, Inc. MN, USA) for 2 hrs, followed by incubation with a secondary antibody (peroxidase conjugated rabbit F(ab')_2 _anti-mouse IgG (Zymed Laboratories, CA, USA)). The positive staining was detected using the Liquid DAB Substrate-Chromogen System (DAKO), followed by counterstaining with Mayers Hematoxylin (Sigma-Aldrich, MO, USA) A matched isotype control, mouse IgG_2B _(clone 20116, MAB004) (Sigma), was used at the same concentration as the primary antibody. Representative pictures were recorded prior to destaining the slides of hematoxylin with 1% HCl in 70% ethanol.

The hematoxylin destained samples were assessed in a blinded fashion using a Leica DC 300F microscope (Leica Microsystems GmbH, Germany) at a magnification of ×200. The positively stained area of the entire tissue section was determined using quantitative imaging software (Leica QWin) with the same threshold used for all sections. The data was expressed as a percentage of total tissue area.

GraphPad 5 (GraphPad Software, Inc. CA, USA) was used for the non-parametric statistical analysis of the percentage of positive staining in tissues. A Mann Whitney test was used to compare the controls with the patient groups and to compare the changes (Δ change) between the 2 patient groups over the season. The Wilcoxon signed rank test was used to compare the changes over the season. A P value of < 0.05 was considered statistically significant. The data are presented in a scatter plot with mean and SEM of the treatment group.

## Results

**Figure 1:**
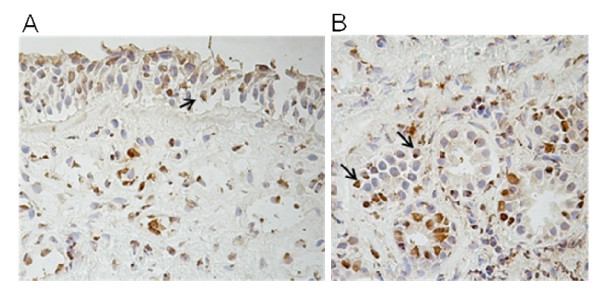
**Immunostaining for osteopontin in nasal biopsies**. Representative pictures of immunostaining for osteopontin in nasal biopsies of allergic rhinitis patients (peak-season, placebo treated) (×400) in epithelium (A) and glands (B). Arrows indicate OPN staining.

Immunohistochemistry revealed clear expression of intracellular OPN in epithelial cells (Figure 1A) and vascular, as well as submucosal gland (Figure 1B) endothelial cells. In most cells, the most prominent staining was observed in the nuclei. The most intense staining was observed in epithelial and endothelial cells, but also in the subepithelial layer to a lesser extent.

Previous studies on these nasal biopsies have shown a clear increase in the number of EG2^+ ^cells over the pollen season and no increase with FP treatment. To determine if the expression of OPN is related to the change in eosinophil numbers previously seen in the AR nasal biopsies, the staining was compared to the EG2^+ ^cell counts previously obtained [2]. The cellular location of the EG2^+ ^staining was distinctly different to that of the OPN staining (data not shown). Additionally, no correlation between the EG2^+ ^cell counts (epithelium or subepithelium) and OPN staining was observed (Spearman R 0.049-0.274).

**Figure 2:**
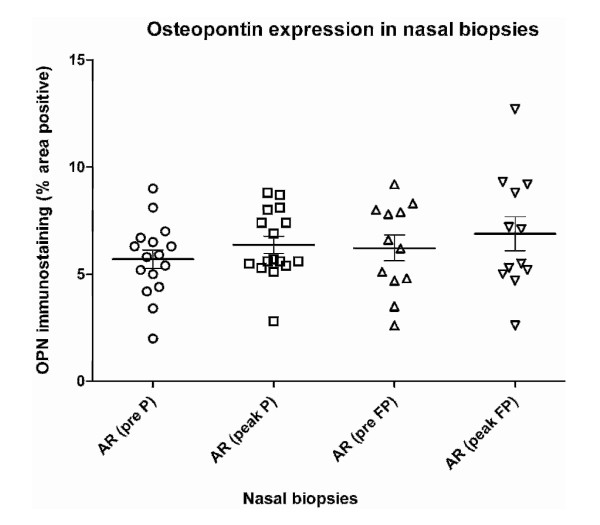
**Percentage of osteopontin stained area in nasal biopsies pre- and peak season**. The percentage of the osteopontin stained area in pre- and peak-season nasal biopsies of allergic rhinitis (AR) patients treated with placebo (P) or fluticasone propionate (FP). The mean and SEM is indicated per group.

The mean OPN expression (% area) was similar in nasal biopsies from healthy controls (4.1 ± 0.8%) compared to pre-season patient samples (5.9 ± 0.4%; p = 0.0706). During the grass pollen season, no significant change in OPN expression was seen in AR patients treated with placebo or FP (Figure 2). Comparison of the difference in OPN expression induced by the grass pollen season revealed no difference between the placebo and FP treated patient groups (p = 0.82).

## Discussion

There is an increased scientific interest in the putative role of OPN in several diseases, including asthma, allergy and rhinosinusitis [7,10,14,15]. In the current study, we have observed clear expression of OPN in the nasal mucosa of both healthy individuals and patients with AR. Natural exposure to pollen did not, however, change the expression of OPN in patients treated with placebo. Lastly, local treatment with a potent nasal glucocorticoid, did not affect the OPN expression in patients with AR during natural exposure to pollen.

While OPN is known to be produced by epithelial and endothelial cells in other healthy tissues [7], our data provides the first example of the distribution in the nasal mucosa of AR patients. The expression of OPN protein in the nasal mucosa of healthy and AR subjects was predominantly in the nucleus of the epithelial and endothelial cells (glandular and vesicular). Similarly, OPN expression has also been seen intracellularly in other nasal biopsies [12], as well as bronchial biopsies [10,11,16].

OPN has been seen to be expressed as a secreted form (s-OPN) and an intracellular form. The s-OPN is commonly measured in different culture supernatants or body fluids. It is extensively modified post-translationally and acts like a Th1 cytokine. Intracellular OPN is less well characterised and has been reported as a translational alternative to s-OPN. It has been shown to be involved in the modulation of cytoskeletal processes [17] as well as in the induction of IFN-α in plasmacytoid dendritic cells [18].

A previous study of the nasal biopsies reported here, looking at the expression of eosinophils [2], showed a clear increase in EG2^+ ^cell numbers induced by a pollen season, which was inhibited with the use of FP. Eosinophils have recently been discussed in relation to OPN [10,13] and as such, the correlation between OPN staining and EG2^+ ^cell numbers was analysed. However, the increase in EG2^+ ^cell numbers induced by season was not mirrored with an increase in OPN expression, confirming that eosinophils are not the only cells producing OPN.

This study shows for the first time that the expression of OPN in the cells of the nasal mucosa of AR patients does not increase over a natural pollen season, although an increase in several cytokines and cells in the nasal mucosa over the pollen season has been reported [2,5,6].

The scope of this study is limited to the intracellular expression of OPN in the nasal mucosa of AR patients. It should be emphasised that although no differences in intracellular OPN expression were observed, this may not be the case for s-OPN. The expression of OPN in the nasal lavage fluid of AR patients should be measured to determine if soluble OPN expression is changed by natural pollen exposure or glucocorticoids. In the lower airways, Takahashi *et al *[10] found that although there was no difference in intracellular OPN expression in the lung, there was a significant increase in the sputum OPN levels between asthmatic and healthy subjects. Conversely, Xanthou *et al *[11] reported an increased intracellular OPN expression in asthmatics, compared to healthy subjects.

It has been previously seen that glucocorticoids are quite effective in inhibiting both the expression of inflammatory cells and clinical symptoms [3] induced by the pollen season. It has previously been shown that FOXP3, GATA-3 [5] and EG2^+ ^cells [2] in the nasal mucosa of AR patients increase during the pollen season and that their expression is suppressed by the nasal glucocorticosteroid FP. Unlike the previous biopsy studies and murine studies [14] the expression of OPN was not inhibited by treatment with FP. This lack of inhibition of OPN expression has also been observed in the serum, bronchial tissue and bronchoalveolar lavage fluid of asthmatics [16]. Erin *et al *[3] has previously shown that FP is effective in inhibiting Th2, but not Th1, cytokine synthesis. OPN has been suggested to act as both a Th1 and Th2 cytokine [8]. The lack of effect by FP on the OPN in the nasal mucosa could suggest that intracellular OPN has a Th1-like role in the nasal mucosa of AR.

## Conclusions

In conclusion, despite the large role that OPN plays in many diseases, including Th2 diseases, like asthma, OPN in allergic rhinitis has been shown here to not be directly involved in the nasal mucosa changes over the pollen season, or in the response to glucocorticoid treatment. This suggests that intracellular OPN in AR patients cannot be used as a marker of disease.

## Competing interests

The authors declare that they have no competing interests.

## Authors' contributions

SO performed the immunohistochemistry and analyses, and drafted the manuscript. KS and CM helped in design of experiment. AB and JO conceived of the study. JO and KS helped draft the manuscript. TP provided the data from a previous eosinophils study. All authors read and approved the final manuscript.
